# Transcutaneous Kilohertz High-Frequency Alternating Current at 10 kHz for Upper-Limb Tremor in People with Parkinson’s Disease: A Double-Blind, Randomized, Crossover Study

**DOI:** 10.3390/jcm13247566

**Published:** 2024-12-12

**Authors:** Juan J. Fernández-Pérez, Juan Avendaño-Coy, Diego Serrano-Muñoz, Filipe Oliveira Barroso, Cristina Montero-Pardo, Beatriz López-Moreno, Alfredo Lerín-Calvo, Juan P. Romero Muñoz, Julio Gómez-Soriano

**Affiliations:** 1 Toledo Physiotherapy Research Group (GIFTO), Faculty of Physiotherapy and Nursing, Universidad de Castilla-La Mancha, 45071 Toledo, Spain; juanjose.fernandez@uclm.es (J.J.F.-P.); diego.serrano@uclm.es (D.S.-M.); julio.soriano@uclm.es (J.G.-S.); 2 Toledo Physiotherapy Research Group (GIFTO), Instituto de Investigación Sanitaria de Castilla-La Mancha (IDISCAM), 45071 Toledo, Spain; 3 Neural Engineering Lab, Cajal Institute, Spanish National Research Council (CSIC), 28002 Madrid, Spain; filipe.barroso@cajal.csic.es (F.O.B.); cristinamontero@cajal.csic.es (C.M.-P.); 4 E.T.S. Ingenieros de Telecomunicación, Universidad Politécnica de Madrid, 28040 Madrid, Spain; 5 “Asociación Parkinson Toledo”, Oslo St, 14, 45003 Toledo, Spain; beatrizcaptoledo@gmail.com; 6 “Grupo de Investigación en Neurociencias Aplicadas a la Rehabilitación” (GINARE), 28045 Madrid, Spain; alerin@neuronrehab.es; 7 Department of Physiotherapy, Faculty of Health Science, The Center for Advanced Studies University La Salle, Universidad Autónoma de Madrid, 28023 Madrid, Spain; 8 Brain Injury and Movement Disorders Neurorehabilitation Group (GINDAT), Institute of Life Sciences, Universidad Francisco de Vitoria, 28223 Madrid, Spain; p.romero.prof@ufv.es; 9 Faculty of Experimental Sciences, Universidad Francisco de Vitoria, 28223 Madrid, Spain; 10 Brain Damage Unit, Beata María Ana Hospital, 28007 Madrid, Spain

**Keywords:** Parkinson’s disease, high-frequency alternating current, tremor, transcutaneous electrical stimulation, handgrip strength, nerve block, randomized controlled trial

## Abstract

**Background/Objectives:** Preclinical studies have evidenced a peripheral nerve blockade with kilohertz high-frequency alternating current (KHFAC) stimulation. It could have a potential effect on aberrant nerve hyperactivity, such as tremor in people with Parkinson’s disease (PwPD). The objective was to investigate the effects of transcutaneous KHFAC at 10 kHz compared with sham intervention on tremor modulation, upper limb motor function, and adverse events in PwPD. **Methods:** This randomized, double-blind, crossover trial included PwPD, who received transcutaneous KHFAC and sham interventions, within a 48 h washout period. Measurements were taken pre-intervention, during, immediately after, and 10 min post-intervention. The main outcomes were rest, postural, and kinetic tremor acceleration. Secondary outcomes were handgrip strength, nine-hole peg test (NHPT), movement onset time, and adverse events. **Results:** Sixteen PwPD were analyzed. Kinetic tremor diminished only in active treatment from baseline at post-intervention (−32.3% (SD 63.3); *p* = 0.03) and 10 min after intervention (−38.9% (SD 60.3); *p* = 0.03). Active treatment showed a greater reduction in kinetic tremor at post-treatment compared to sham (−58.7% SD 123; *p* = 0.055) close to reaching statistical significance. Only active intervention diminished movement onset time at post-intervention (−26.9% (SD 28.3); *p* = 0.04). Active intervention diminished handgrip strength compared to sham intervention during the stimulation (−6.6% (SD 10.0); *p* = 0.02). No relevant adverse effects were reported. **Conclusions:** KHFAC stimulation at 10 kHz appeared safe and showed potential benefits for reducing kinetic tremor in PwPD. The transient reduction in grip strength suggested an effect on alpha-motoneurons. However, further studies with larger sample sizes are necessary to confirm these findings.

## 1. Introduction

Parkinson’s Disease (PD) is the second most common progressive neurodegenerative disorder, defined by both motor and non-motor symptoms, reflecting its systemic nature [1,2]. Key motor symptoms, including bradykinesia, rest tremor, and rigidity, are central to its diagnosis, according to contemporary diagnostic criteria [2]. Among these, parkinsonian tremor is one of the hallmark motor symptoms, defined by involuntary oscillatory movements at 4–6 Hz, typically asymmetrical and predominantly occurring at rest [3]. Postural and kinetic tremors are less common but affect up to one-third of patients [4]. Overall, about 80% of people with Parkinson’s disease (PwPD) experience some form of tremor [4], making it one of the most prevalent symptoms [5]. Additionally, non-motor symptoms such as cognitive, behavioral, and autonomic dysfunctions further distinguish PD from atypical parkinsonisms, enhancing its complexity [6,7].

Pharmacological treatment is the first-line approach for managing PD symptoms, primarily through dopamine replacement therapies [8]. Notably, about 39% of PwPD have a dopamine-resistant resting tremor (RT) [9], needing new approaches. While invasive options such as magnetic resonance-guided focused ultrasound, deep brain stimulation, and stereotactic radiosurgery (Gamma Knife) have proven their effectiveness, these interventions are associated with higher costs and greater risks of serious adverse effects compared to non-invasive treatments [10,11]. Given the limitations of pharmacological and invasive treatments, non-invasive electrical stimulation has emerged as a promising alternative for tremor modulation in PwPD [12,13]. Various forms of low-frequency (between 20 and 250 Hz) transcutaneous electrical stimulation have been applied on the upper limb, without significant adverse effects. Nevertheless, the effectiveness of this stimulation intervention on tremor modulation is heterogeneous [12,13].

Recent research on peripheral electrical stimulation with kilohertz high-frequency alternating current (KHFAC) suggests its potential to modulate motor hyperactivity. Preclinical studies have demonstrated that KHFAC stimulation can induce a rapidly reversible axonal block without causing nerve damage [14]. This functional blockade of the axon may be attributed to electrical stimuli application at supraphysiological frequencies exceeding 1000 Hz [15]. Consequently, this peripheral nerve block could have a potential effect on neurological conditions characterized by aberrant nerve hyperactivity, such as tremor in people with Parkinson’s disease (PwPD).

In healthy participants, both transcutaneous and percutaneous KHFAC stimulation of peripheral nerves has been shown to reduce maximal isometric strength at frequencies of 10 kHz [16,17,18] and 20 kHz [16,19], without causing significant adverse effects. These changes in motor activity provide an indirect measure of α- motoneurons modulation. Recently, a case study reported, for the first time in humans, a complete motor blockade induced by KHFAC stimulation at 20 kHz using a cuff electrode applied directly to the nerve [20]. This demonstration of nerve block via KHFAC is a significant advancement toward the clinical application of these currents in patients with pathological motor hyperactivity.

Previous evidence from healthy volunteers has demonstrated that transcutaneous KHFAC stimulation at 10 kHz and 20 kHz can modulate motor activity. However, no studies to date have examined the effects of KHFAC in populations with neurological disorders. This study is the first to investigate transcutaneous KHFAC stimulation in individuals with neurological conditions. We hypothesized that motor activity modulation via transcutaneous KHFAC stimulation could reduce tremor in PwPD. The primary objective was to assess the effects of 10 kHz KHFAC stimulation on tremor compared to sham in PwPD. Secondary objectives were to evaluate the effect on upper limb dexterity, movement onset time, isometric grip strength, perceived patient improvement, adverse events, and the success of blinding.

## 2. Materials and Methods

### 2.1. Study Design and Register

A randomized, double-blind, sham-controlled, crossover study involving PwPD was conducted. The CONSORT 2010 checklist revised for the crossover trial was followed [21]. Participants received two interventions: active stimulation using KHFAC at 10 kHz, and sham intervention. The order of the interventions was randomized by a computer-generated sequence at www.randomizer.org (accessed on 15 December 2023). The randomization process was managed by an independent researcher (JGS) who was not involved in participant evaluations or data analysis. The intervention sequence for each participant was sealed in opaque, sequentially numbered envelopes to maintain blinding. Only the investigators administering the intervention (ALC, JAC, AGA) were aware of the allocation, while participants and assessors remained blinded. A washout period of at least 48 h was implemented between sessions, since the KHFAC effects are rapidly reversible, within a few minutes after the intervention [14]. For this reason, this period is enough to avoid the carry-over effects.

Before the initiation of the study, ethical approval was obtained from the Ethics Committee of the University Hospital of Toledo (number: 717; date of approval: 26/05/2021). The study was registered in clinicaltrial.gov (reference number: NCT06247423). Informed consent was given before the inclusion of the participants, according to the Declaration of Helsinki [22].

### 2.2. Participants and Eligibility Criteria

The sample was recruited from “Asociación Parkinson de Toledo”, “Asociación de Parkinson de Talavera de la Reina (ADEPAR)”, and “Asociación de Parkinson Fuenlabrada (ASPARFU)”, Spain. The experimental phase was conducted in the laboratory at the University of Castilla-La Mancha in Toledo, Spain, under controlled sound conditions and a stable room temperature was maintained between 22 and 26 °C. All participants were in an off state during data collection and interventions.

Inclusion criteria were as follows: participants with PD diagnosed by a specialized neurologist, exhibiting tremor as defined by the Movement Disorder Society consensus statement [23], sufficient cognitive function to comprehend all measurement procedures, tolerance for electrical stimulation, and willingness to participate in the study. Exclusion criteria included any pharmacological or invasive treatments for tremor suppression (including tremor medication intake within at least 9 h prior to each intervention), altered sensitivity near the stimulation site, recent trauma or surgeries affecting measurement accuracy, presence of pacemakers or other implanted electrical devices, tattoos or other external agents at the treatment site, and comorbidities or other pathologies impacting tremor or coordination.

### 2.3. Intervention

The stimulation was delivered utilizing the same stimulator (Myomed 932, Enraf-Nonius, Delft, The Netherlands) for both interventions. Self-adhesive gel electrodes (Protens, 50 × 100 mm) were positioned on the upper limb exhibiting more tremor. Specifically, one electrode was situated on the medial aspect of the arm, 8–10 cm above the medial epicondyle (over the median and ulnar nerves). The other electrode was affixed on the lateral aspect of the arm 4–6 cm above the lateral epicondyle (over the radial nerve).

The active treatment session involved the application of stimulation via an unmodulated alternating sinusoidal current, with a frequency of 10 kHz for 20 min. Following electrode placement, the current intensity was progressively increased with a 30 s ramp-up, until reaching a “strong but comfortable tingling sensation”, just below the motor threshold [16,19]. Continuous adjustments to intensity were implemented to maintain the tingling sensation throughout the session. Whenever the participant’s sensation of tingling diminished, the stimulation intensity was increased. However, if muscle contractions were observed by the researchers or reported by the participants, or if the tolerance threshold was reached, the intensity was reduced accordingly.

In the sham intervention, the same device and electrode positioning were employed as in the active protocol. However, the intensity was progressively augmented with a 30 s ramp-up until reaching the motor threshold. Afterward, it was reduced to 0 mA, with a 30 s ramp-down, and maintained at that level throughout the intervention [19,24].

### 2.4. Measures

All the measures were taken in the same position as the intervention on the upper limb with more tremor. Participants were seated in a chair, with the forearm exhibiting the most prominent tremor resting on an armrest, the shoulder in slight abduction, and the elbow flexed at 90 degrees.

Anthropometric, demographic, and clinical data were collected before the intervention by a researcher (BL) who was not involved in the intervention. These variables were age, sex, weight, height, body mass index, time since PD diagnosis and medication intake, medication in milligrams per day, previous history of injuries, family history of tremor, dominant hand, and upper limb with the highest tremor. The daily levodopa equivalent dose was obtained using the calculator at https://www.parkinsonsmeasurement.org/ (accessed on 5 January 2024) and an updated review of levodopa dose equivalency for non-included dopaminergic medication [25]. To evaluate motor and non-motor PD aspects, the Movement Disorder Society–Unified Parkinson’s Disease Rating Scale (MDS-UPDRS) Part II and III scores [26], and classification by Hoehn and Yahr were collected [27]. Moreover, PD motor predominant subtypes were extracted from the MDS-UPDRS: (1) tremor dominant; (2) akinetic-rigid; and (3) mixed [28].

Tremor-related outcomes, nine-hole peg test (NHPT), and handgrip strength measures were taken at four time-points: pre-intervention, during the intervention (at 10 min since stimulation onset), immediately after stimulation (20 min), and 10 min post-stimulation. The Patient Global Impression of Improvement (PGI-I) and blinding success were assessed after each intervention, while adverse effects were recorded at the end and 24 h after both interventions.

#### 2.4.1. Main Outcomes

Tremor-related outcomes—including RT, postural tremor (PT), and kinetic tremor (KT)—were recorded using the Wireless Biofeedback System (Trigno^®^, SP-W06-017 Delsys Inc., Natick, MA, USA). This system uses inertial measurement units (IMUs) to register acceleration (G-force) with a sampling rate of 148 Hz. The adhesive-mounted sensor was affixed to the abductor pollicis brevis. Skin preparation and electrode placement followed the guidelines outlined by the Surface Electromyography for the Non-Invasive Assessment of Muscles “SENIAM” (available at http://www.seniam.org/ (accessed on 15 January 2024)). Following data collection, the average acceleration across three axes was processed using a MATLAB script to calculate the area under the curve of the power spectral density (PSD). A 2 s Hamming window with 50% overlap was applied to compute the PSD, which was subsequently integrated over the 3–9 Hz frequency range.

For recording RT, participants were instructed to place their forearm on the armrest, with the wrist unrestricted and the elbow flexed. They were asked to count as fast as they could from 100 to 0 for a minimum of 90 s or until the tremor was evoked [3]. The last 30 s window was taken for the posterior analysis. The PT was assessed with participants maintaining a 90° flexed shoulder posture, extended forearms, and neutral wrist position for 30 s [29]. The central 15 s window was taken for a posterior analysis. The KT was evaluated by instructing participants to grasp a cup by the handle, oriented toward the arm with a more prominent tremor. Afterward, participants bring the cup to their mouth as if drinking, maintaining the position for three seconds, and returning it to its initial position [30]. The complete movement was analyzed.

#### 2.4.2. Secondary Outcomes

The NHPT was used to assess upper limb function and finger dexterity. Participants placed and removed nine plastic pegs individually in a container with vertically oriented slots, using one hand. The simultaneous handling of multiple pegs was not allowed. Timing began when the participant touched the first peg and ended when the last peg was placed into the container, with the total time recorded in seconds [31]. The NHPT has exhibited excellent intra-rater reliability (ICC = 0.88 dominant hand; ICC = 0.91 non-dominant hand) [31] and inter-rater reliability (ICC > 0.99) in previous studies [32]. Due to the impaired ability of individuals with PD to initiate movements and reach for objects, the outcome measure of onset time was incorporated into the evaluation. This outcome measured the time from the auditory cue “go” until the participant reached the first peg, as recorded by a stopwatch [33].

Maximum isometric handgrip strength was gauged using a grip dynamometer (Grip Strength Dynamometer T.K.K. 5401 GRIP-D Takei Scientific Instruments CO., Ltd. Shinagawa-ku, Tokyo, Japan), which showed to be a reliable and valid instrument [34]. Participants exerted the maximum grip strength for 3 s, performing three repetitions with at least 15 s of rest [35]. The resulting force was calculated by averaging the three measurements in kilograms-force (Kgf).

Current-induced sensations and skin adverse effects were collected via a questionnaire, including ‘Yes/No’ responses to assess redness, pain, swelling, heat, coldness, numbness, loss of strength, heaviness, tingling, or other sensations. The perceived unpleasantness level during the intervention was evaluated using a visual analog scale, where 0 cm corresponded to ‘none’ and 10 cm to ‘the maximum possible’, similar to a previous study of our research group [24].

The Patient Global Impression of Improvement (PGI-I) was administered after each intervention to determine the patient’s subjective improvement level. Participants were asked: “How do you feel your tremor after the intervention?”. This Likert scale ranged from 1 ‘much better’ to 7 ‘much worse’ [36].

The success of participant and evaluator blinding was assessed post-intervention [37]. Participants and assessor were asked ‘What type of treatment do you believe you or the participant received?’ with five response options: (1) ‘I firmly believe I/he/she received an experimental treatment’; (2) ‘I somewhat believe I/he/she received an experimental treatment’; (3) ‘I firmly believe I/he/she received a placebo’; (4) ‘I somewhat believe I/he/she received a placebo’; (5) ‘I don’t know, I don’t answer’.

### 2.5. Statistical Analysis

Before analysis, outliers exceeding ±2 standard deviations from the mean were identified and removed through a listwise deletion method [38,39]. Following this, data were normalized from the baseline mean values. The raw data of tremor and mean and standard deviation of each outcome can be found in Supplementary Tables S1 and S2.

The Shapiro–Wilk test was employed to assess the normality of the outcomes. For within-intervention analysis from baseline, a repeated measures analysis of variance (ANOVA) with Bonferroni post-hoc correction was conducted for maximal isometric hand-grip strength and NHPT onset time. When the assumption of sphericity was violated, the Greenhouse–Geisser correction was applied. The Friedman test was utilized for outcomes that did not follow a normal distribution, such as tremor-related outcomes and NHPT total time, using the Wilcoxon signed-rank test for within-intervention pairwise comparisons.

Between-intervention comparations of change from baseline were assessed using a paired t-test for normally distributed data, including effect sizes with Cohen’s d [40]. The Wilcoxon signed-rank test was applied to non-normally distributed variables, obtaining the effect size using Rosenthal’s r [41]. Fisher’s exact test was used to analyze binary outcomes such as stimulation sensations and skin-related adverse events, while continuous variables like unpleasantness, current intensity, and ordinals like the PGI-I were analyzed using the Wilcoxon signed-rank test. A *p*-value of less than 0.05 was considered statistically significant. Data were expressed as the mean and standard deviation (SD), and statistical analyses were performed using IBM SPSS Statistics version 29.0.1.0. (IBM Corp., Armonk, NY, USA) 

The blinding outcome variables, James’ Blinding Index (BI) [37] and Bang’s BI [42] were calculated using Stata v15.0 (Stata Corp., College Station, TX, USA). James’ BI assesses the overall success of blinding in randomized control trials, while Bang’s BI evaluates the blinding status within each trial arm independently. James’ BI ranges from 0 to 1, where 0 indicates a total lack of blinding, 1 indicates complete blinding, and 0.5 indicates random blinding. In this study, a lack of blinding was inferred if the upper bound of the confidence interval (CI) was below 0.5. Bang’s BI ranges from −1 to 1, with 0 representing complete random blinding. This index can be interpreted as the proportion of unblinding in each arm. If the one-sided CI did not include 0, the study was considered to lack blinding, whereas if the one-sided CI does not cover 0 and the BI is negative, it indicates an opposite guess.

## 3. Results

### 3.1. Participants and Current Intensity

**Figure 1:**
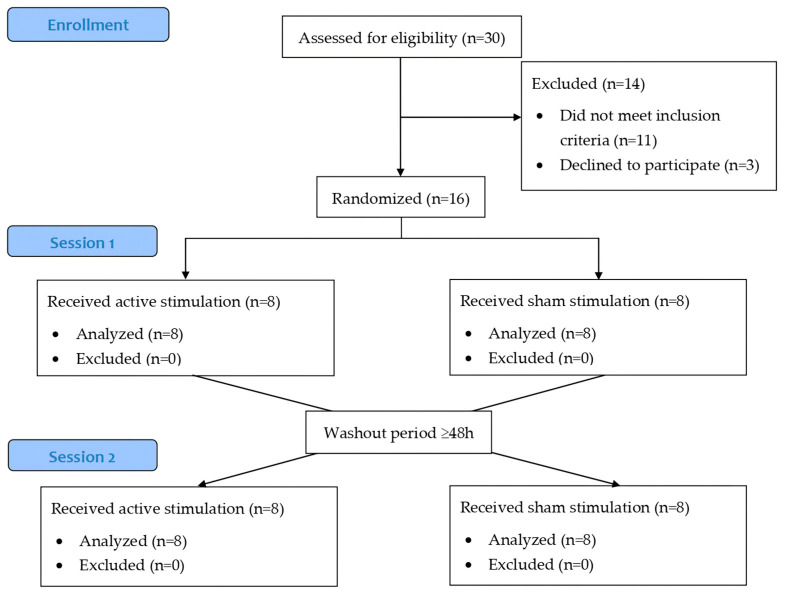
Consolidating standards of reporting trials (CONSORT) flowchart.

Thirty PwPD were initially screened for the study. Eleven participants were excluded for not meeting the inclusion criteria, and three declined to participate (Figure 1). Finally, sixteen participants were randomized, 38% were women and 6% were left-handed, with a mean age of 68.9 years (SD 8.3). Regarding predominant motor subtypes, 31% of participants were tremor-dominant, 56% akinetic-rigid, and 13% were mixed. All participants completed both sessions. Further demographic and baseline clinical data are detailed in Table 1. Regarding current intensity during active intervention, the initial value was 82.3 mA (SD 47.9), which increased to 105.0 mA (SD 52.9) at the end of stimulation. The Wilcoxon signed-rank test indicated a significant difference (22.8 mA (SD 30.2; *p* = 0.002)).

### 3.2. Main Outcomes

#### 3.2.1. Resting Tremor

Detailed information about within and between-intervention comparisons for tremor outcomes is shown in Table 2. Four participants’ values for this outcome were identified as outliers and removed, including n = 12 participants for the RT analysis. The Friedman test showed no significant within-intervention differences in either the active (χ^2^ = 2.6; *p* = 0.47) or sham interventions (χ^2^ = 3.5; *p* = 0.32). The Wilcoxon signed-rank test showed no significant within-intervention differences from baseline in both interventions. Moreover, no significant differences were found between interventions at any time-point (Table 2).

#### 3.2.2. Postural Tremor

Four participants´ values for this outcome were identified as outliers and removed, including n = 13 participants for the PT analysis. The Friedman tests showed non-significant differences in active (χ^2^ = 6.5; *p* = 0.09) or sham intervention (χ^2^ = 4.5; *p* = 0.21). However, the Wilcoxon signed-rank test revealed a significant within-intervention difference from baseline at 10 min post-intervention (−56.5% (SD 122.7); *p* = 0.01) in active treatment and at post-intervention (−37.0% (SD 103.1); *p* = 0.046) in the sham treatment. No significant differences between interventions were identified at any time-point (Table 2).

#### 3.2.3. Kinetic Tremor

**Figure 2:**
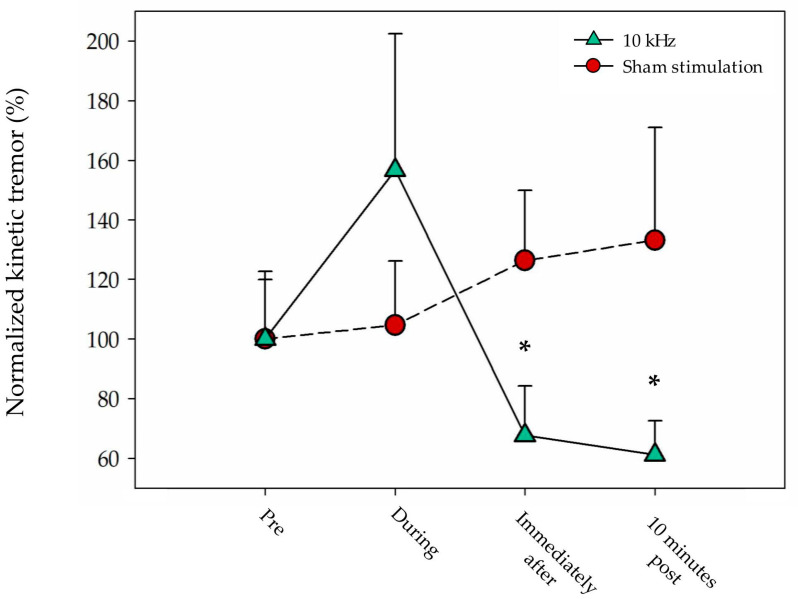
Normalized kinetic tremor (%) across each intervention time-point. Values are presented as the mean and standard error of the mean (error bars). Asterisks (*) mark statistically significant within-intervention differences when compared to pre-intervention values (*p* < 0.05).

Three participants´ values for this outcome were identified as outliers and removed, including n = 13 participants for the KT analysis. The Friedman tests showed significant differences in active treatment (χ^2^ = 9.1; *p* = 0.028), but not in the sham treatment (χ^2^ = 3.2; *p* = 0.36). The Wilcoxon signed-rank test revealed a significant within-intervention difference from baseline in active treatment immediately after the intervention (−32.3% (SD 63.3); *p* = 0.03), and at 10 min post-intervention (−38.9% (SD 60.6); *p* = 0.03), without changes in sham treatment (Table 2). Furthermore, the comparison of the change between interventions showed a greater reduction in KT in the active group than in the sham group at post-treatment at the limit of statistical significance (−58.7% (SD 123.0); *p* = 0.055) (Table 2, Figure 2).

### 3.3. Secondary Outcomes

#### 3.3.1. Maximum Isometric Handgrip Strength

All participants (n = 16) were analyzed for this outcome. The repeated measure ANOVA showed no significant differences over time (F = 1.7; *p* = 0.21). Although non-significant within-intervention differences from baseline were found, the active group showed a trend toward reduced strength, whereas the sham group exhibited a trend toward increased strength (see Table 2). The comparison of the change between interventions showed a greater reduction in grip strength in the active group than in the sham during the intervention (−6.6% (SD 10.0); *p* = 0.01), with a moderate effect size (Cohen’s d = −0.66; 95%CI [−1.2, −0.1]) (Table 2).

#### 3.3.2. Nine-Hole Peg Test—Onset and Total Time

Four participants´ values for movement onset time of NHPT were identified as outliers and removed; n = 12 participants were analyzed. The repeated measure ANOVA reported significant within-intervention differences over time (F = 6.1; *p* = 0.002). A significant decrease in movement onset time was observed only in the active intervention at 10 min post-intervention (−26.9% (SD 28.3); *p* = 0.04). No significant differences between interventions were observed at any time-point (Table 2).

Following the identification and exclusion of two outliers, the final sample for NHPT total time analysis was n = 14. The Friedman test showed a significant effect within the active intervention (χ^2^ = 10.0; *p* = 0.02), whereas no significant differences were observed in the sham intervention (χ^2^ = 3.4; *p* = 0.33). The Wilcoxon signed-rank test showed non-significant within-intervention differences from baseline in both interventions. No significant differences between interventions were observed (Table 2).

#### 3.3.3. Patient Global Impression of Improvement

All participants n = 16 were analyzed for this outcome. Similar scores were reported for PGI-I in active (3.37 score (SD 1.0)) and sham (3.47 score (SD 0.9)) interventions. The Wilcoxon signed-rank test indicated no significant differences between interventions (−0.1 score (SD 0.9); *p* = 0.76).

#### 3.3.4. Current-Induced Sensations and Skin Adverse Events

All participants n = 16 were analyzed for these outcomes. Detailed data regarding perceived sensations and skin adverse events are summarized in Table 3. Tingling, numbness, and heaviness were the most frequently reported sensations during stimulation. No significant differences between interventions were observed. Mild adverse skin events, such as erythema, were localized beneath the electrodes. They were observed immediately following stimulation and resolved within 24 h, except for one case due to electrode misplacement over a skin fold. Greater perceived unpleasantness was found in the active intervention (0.9 cm (SD 1.7); *p* = 0.03) compared to the sham intervention.

#### 3.3.5. Blinding Success

Table 4 displays the outcomes of the blinding of all participants (n = 16) and assessor. The overall analysis via James’ index determined the lack of blinding of both the assessor and the participants. The blinding analysis by the interventions using Bang’s BI revealed a lack of blinding in the active intervention of the participants and the assessor. However, this BI also revealed blinding of participants (opposite guess) in the sham intervention, while the assessor showed a lack of blinding.

## 4. Discussion

This is the first study to evaluate the effects of transcutaneous 10 kHz KHFAC stimulation on tremor modulation in PwPD. Although active interventions showed greater tremor reductions in most time-points, no statistically significant differences were observed between interventions for RT and PT. However, KT showed significant inhibition immediately after and 10 min post-intervention with 10 kHz stimulation, an effect not observed with sham stimulation, with a near-significant difference between interventions (*p* = 0.055). Handgrip strength was the only outcome that differed significantly between interventions, with a greater reduction observed during the 10 kHz stimulation intervention. Nevertheless, it is important to note that this change did not modify significantly the fine motor dexterity and upper limb coordination, measured with NHPT total time. Moreover, a significant within-intervention difference in NHPT onset time was found only in the active intervention at 10 min post-intervention. Regarding safety, although higher unpleasantness in the active stimulation was reported, it remained low (1.7 out of 10). In any case, adverse effects did not differ significantly from both interventions, proving that KHFAC at 10 kHz is a safe stimulation intervention. 

The rationale for this study was to apply 10 kHz electrical stimulation to induce a motor nerve block to disrupt the pathological tremor in PwPD. The frequency of 10 kHz was selected because it is effective in partially blocking the motor activity of the median nerve in healthy subjects [17,18]. Furthermore, a recently published case report study has evidenced a motor nerve block to inhibit spasticity induced by an implanted cuff electrode stimulating at 20 kHz in a subject with cerebral palsy [20]. The percutaneous application of 20 kHz also evidenced a partial motor nerve block that was not shown when applying 10 kHz [16]. These findings, combined with the absence of tremor reduction observed in our study, support the need for future studies employing 20 kHz stimulation.

Even though a reduction of about 33% was observed for rest and postural tremors during the application of 10 kHz, these results were not statistically significant, probably due to high baseline tremor variability and inconsistent effects. Regarding KT, a significant reduction was observed immediately after the stimulation (32%) and after 10 min (39%), reaching almost a significant difference (*p* = 0.055) when compared to sham immediately after the stimulation. Other approaches that have used peripheral electrical stimulation with low frequencies (10–150 Hz) on tremor-related conditions have reported a reduction of tremor from 35% to 60% [13]. However, most of these studies have a quasi-experimental pre-post design without including a control group [13]. Only one study has included a sham stimulation group to adequately blind and control the outcomes [43]. The authors applied low-frequency electrical stimulation above the motor threshold to hand muscles via a tremor glove, achieving a 40–60% reduction in intractable tremor. Future studies should validate the efficacy of various stimulation paradigms on pathological tremor compared to an appropriate sham condition.

The observed reduction in handgrip strength during the active intervention (−5.1% (SD 10.5)) did not exceed the minimal detectable change, which was established at 13% for PwPD [44]. However, this reduction is consistent with previous studies on neurologically intact participants, whose authors applied KHFAC stimulation in upper limb peripheral nerves at frequencies between 5 and 20 kHz [16,17,18,19]. Transcutaneous KHFAC at 10 kHz led to strength reductions of 14–40% in maximal and submaximal isometric strength during stimulation [17,18]. Another study applying KHFAC at 20 kHz observed a decrease of 12% in handgrip strength during stimulation [19]. Percutaneous KHFAC stimulation at 10 and 20 kHz resulted in post-stimulation reductions in maximal isometric index finger strengths of 8.5% and 12%, respectively [16]. The smaller magnitude of change observed in this study, compared to reductions reported in studies with healthy participants, may be due to the generally lower isometric strength typically found in PwPD, which could mask more pronounced effects.

Our initial hypothesis proposed that a partial nerve block induced by 10 kHz KHFAC would interfere with fine motor control and hand dexterity, as measured by the NHPT. However, it remains unclear whether there is any direct relationship between the induced nerve block and fine motor control, or if the lack of changes in NHPT total time across both interventions is due to an ineffective motor nerve block, insufficient to alter tremor, and only slightly reduced strength. For instance, the study published by Hu et al. [45] showed a reduction in tremor induced by transcutaneous electrical stimulation that was accompanied by an increase in the movement time of a task executed with the upper limb, without interrupting the voluntary movement control in PwPD.

Additionally, NHPT onset time was measured to indirectly assess movement initiation impairments typically observed in PwPD. A significant reduction in onset time was observed 10 min after the 10 kHz active stimulation, albeit not in sham stimulation. This effect suggests a possible influence of KHFAC on supraspinal mechanisms involved in movement initiation and motor execution [33]. However, future studies should be designed using validated outcomes to address the specific effect of KHFAC on motor processing [46].

Previous studies in the field of transcutaneous KHFAC suggested the clinical implications of nerve blocks in the field of chronic pain or motor disorders [14]. This is the first study in a population with neurological disorders aimed to validate the use of 10 kHz KHFAC to modulate pathological tremor. Although the stimulation was well-tolerated and adverse effects were mild and comparable to those associated with other forms of transcutaneous electrical stimulation, such as TENS [47], the lack of a clear efficacy in tremor reduction limits its potential for clinical translation. Future studies should prioritize optimizing stimulation parameters, as stimulation at 20 kHz may produce greater effects [16].

Our findings suggest that KHFAC stimulation may serve as a safe intervention capable of modulating peripheral motor activity. This approach could be feasible for other neurological disorders characterized by pathological motor hyperactivity, such as hypertonia and rigidity. The observed reduction in KT further underscores its relevance and potential utility in individuals with conditions associated with this outcome, such as essential tremor [48]. Additionally, the significant effects on postural and kinetic tremor observed 10 min after stimulation suggest the potential value of exploring repetitive stimulation sessions with an extended follow-up period

This study has some relevant limitations. Firstly, there was considerable inter-individual variability in tremor severity, which exceeded our expectations. This variability reduces the statistical power and could lead to a type II error, masking some real effects. Future studies should consider this variability in sample size calculations. Secondly, although this study employed a double-blind, sham-controlled design, the lack of blinding success among participants and evaluators may have introduced detection bias. This blinding method is commonly used with other forms of electrical stimulation [49,50] and is generally effective [51]. However, new blinding approaches may be required for this type of stimulation. Finally, the difference observed in NHPT onset time in the 10 kHz stimulation group could have been influenced by using a manual stopwatch. Future research should assess the effects of KHFAC on impaired movement initiation using validated measurement techniques.

## 5. Conclusions

Transcutaneous KHFAC at 10 kHz did not demonstrate superior modulation of resting, postural, or kinetic tremors in PwPD compared to sham intervention. However, active stimulation significantly reduced KT immediately after and 10 min post-intervention, and notably decreased handgrip strength during stimulation relative to sham, suggesting a partial block effect on α-fibers. Furthermore, the intervention was safe and well-tolerated. The absence of significant differences between groups, despite being close to statistical significance for kinetic tremor, should be interpreted with caution due to the methodological limitations of this study. The low statistical power resulting from the small sample size and the high variability in tremor outcomes must be considered when interpreting the findings. Future studies should focus on larger sample sizes, optimized stimulation parameters, and refined protocols to improve clinical outcomes.

## Figures and Tables

**Table 1:** Participant characteristics.

Demographic Data (n = 16)	
Outcomes (Units)	Mean (SD)
Gender (female/male)	10/6
Age (years)	68.9 (8.3)
BMI (kg/m^2^)	26.9 (4.1)
Dominant hand (right/left)	15/1
**Clinical data (n = 16)**	
Time since PD onset (years)	5.5 (4.6)
Hand with more tremor (right/left)	7/9
Levodopa equivalent dose (mg)	751.8 (440.6)
MMSE	28.7 (2.8)
H&Y stages (I/II/III)	4/7/5
*MDS-UPDRS*	
Part I—(fatigue)	1.1 (1.0)
Part II	14.8 (6.5)
Part III	32.1 (7.8)
*Predominant tremor*	
Rest tremor n (%)	14 (88)
Mixed tremor n (%)	2 (12)
*Predominant Motor subtype*	
Akinetic-rigid n (%)	9 (56)
Tremor-dominant n (%)	5 (31)
Mixed n (%)	2 (13)

BMI: Body Mass Index, MDS-UPDRS: Movement Disorder Society–Unified Parkinson Disease Rating Scale, MMSE: Mini-Mental State Examination, PD: Parkinson’s disease, SD: Standard Deviation.

**Table 2:** Within and between-intervention differences from pre-intervention values in percentage of change.

Outcomes (Units)		10 kHz	Sham		
	Time	Within-Intervention Change from Pre Mean (SD) [*p*-Value]	Within-Intervention Change from Pre Mean (SD) [*p*-Value]	Between-Intervention Change Mean (SD) [*p*-Value]	Effect Size [CI 95%]
Rest tremor (%)	∆ During	−32.9 (128.5) [0.45] ^c^	−6.4 (88.7) [0.24] ^c^	−26.5 (100.8) [0.59] ^c^	−0.15 ^e^
	∆ Post	2.1 (77.5) [0.53] ^c^	−23.1 (57.9) [0.16] ^c^	25.2 (97.0) [0.06] ^c^	0.54 ^e^
	∆ Post10	−22.0 (74.2) [0.16] ^c^	174.3 (467.2) [0.37] ^c^	−196.3 (530.8) [0.43] ^c^	−0.23 ^e^
Postural tremor (%)	∆ During	−33.0 (120.9) [0.42] ^c^	−6.1 (107.8) [0.12] ^c^	−26.9 (122.3) [0.97] ^c^	−0.01 ^e^
	∆ Post	−41.0 (133.8) [0.20] ^c^	**−37.0 (103.1) [0.046] ^c^**	−4.0 (109.3) [0.45] ^b^	−0.04 [−0.6, 0.5] ^d^
	∆ Post10	**−56.5 (122.7) [0.01] ^c^**	−40.7 (92.1) [0.17] ^c^	−15.8 (79.1) [0.24] ^b^	−0.20 [−0.7, 0.4] ^d^
Kinetic tremor (%)	∆ During	56.7 (166.7) [0.15] ^c^	4.7 (67.1) [0.42] ^c^	52.1 (177.4) [0.51] ^c^	0.18 ^e^
	∆ Post	**−32.3 (63.3) [0.03] ^c^**	26.4 (114.2) [0.35] ^c^	−58.7 (123.0) [0.055] ^b^	−0.48 [−1.0, 0.1] ^d^
	∆ Post10	**−38.9 (60.6) [0.03] ^c^**	33.2 (133.8) [0.51] ^c^	−72.1 (166.2) [0.22] ^c^	−0.34 ^e^
Handgrip strength (%)	∆ During	−5.1 (10.5) [0.42] ^a^	1.5 (6.8) [1.00] ^a^	**−6.6 (10.0) [0.01] ^b^**	**−0.66 [−1.2, −0.1] ^d^**
	∆ Post	−1.8 (12.3) [1.00] ^a^	1.2 (6.6) [1.00] ^a^	−3.1 (12.0) [0.16] ^b^	−0.26 [−0.8, 0.2] ^d^
	∆ Post10	1.0 (9.4) [1.00] ^a^	2.4 (10.1) [1.00] ^a^	−1.4 (13.7) [0.34] ^b^	−0.10 [−0.6. 0.4] ^d^
NHPT onset time (%)	∆ During	7.9 (33.9) [1.00] ^a^	9.2 (21.4) [0.97] ^a^	−1.3 (35.2) [0.45] ^b^	−0.04 [−0.6, 0.5] ^d^
	∆ Post	−12.4 (48.0) [1.00] ^a^	−13.8 (30.7) [0.88] ^a^	1.5 (56.5) [0.47] ^b^	0.03 [−0.5, 0.6] ^d^
	∆ Post10	**−26.9 (28.3) [0.04] ^a^**	−14.6 (33.7) [0.97] ^a^	−12.4 (36.2) [0.13] ^b^	−0.34 [−0.9, 0.2] ^d^
NHPT total time (%)	∆ During	17.5 (34.6) [0.06] ^c^	8.2 (15.8) [0.12] ^c^	9.3 (42.6) [0.41] ^c^	0.22 ^e^
	∆ Post	4.6 (16.9) [0.10] ^c^	−0.4 (22.6) [0.80] ^c^	5.0 (29.1) [0.27] ^b^	0.17 [−0.4, 0.7] ^d^
	∆ Post10	−4.9 (12.5) [0.17] ^c^	−1.1 (14.9) [0.73] ^c^	−3.8 (20.7) [0.25] ^b^	−0.18 [−0.7, 0.3] ^d^

CI: confidence interval, NHPT: nine-hole peg test, Post: immediately after the intervention. Bold numbers represent a significant difference (*p* < 0.05). ^a^ Repeated measure ANOVA with Bonferroni correction; ^b^ T-student paired sample test; ^c^ Wilcoxon signed-rank test; ^d^ Cohen’s d; ^e^ Rosenthal’s r.

**Table 3:** Stimulation-related sensations, adverse skin events, and unpleasantness reported in each intervention.

Outcomes	10 kHz (n = 16)	Sham (n = 16)	*p* Value
Tingling n (%)	16 (100)	16 (100)	1.00 ^a^
Numbness n (%)	4 (25.0)	1 (6.3)	0.33 ^a^
Heaviness n (%)	2 (12.5)	1 (6.3)	1.00 ^a^
Pain n (%)	2 (12.5)	1 (6.3)	1.00 ^a^
Warmth n (%)	2 (12.5)	0 (0.0)	0.48 ^a^
Erythema n (%)	4 (25.0)	0 (0.0)	0.10 ^a^
Unpleasantness. VAS cm Mean (SD)	**1.7 (1.9)**	**0.8 (0.9)**	**0.03 ^b^**

n: number of participants, PGI-I: Patient Global Impression of Improvement, SD: Standard Deviation, VAS: Visual Analogue Scale. Bold numbers indicate significant differences between interventions. ^a^: Fisher’s exact test (two-tailed); ^b^: Wilcoxon signed-rank test.

**Table 4:** Blinding indexes assessment of participants and assessor.

Participants Blinding (n = 16)				
Methods	Index	*p*-Value	95% CI	Conclusion
James’ BI	0.38	0.03	0.28 to 0.48	Unblinded
Bang’s BI-Active/2 × 5	0.81	*p* < 0.001	0.73 to 0.92	Unblinded
Bang’s BI-Placebo/2 × 5	−0.40	0.98	−0.73 to −0.08	Opposite guess
**Assessor blinding (n = 16)**				
**Methods**	**Index**	***p*-Value**	**95% CI**	**Conclusion**
James’s BI	0.21	*p* < 0.001	0.09 to 0.34	Unblinded
Bang’s BI-Active/2 × 5	0.53	*p* < 0.001	0.42 to 0.64	Unblinded
Bang’s BI-Placebo/2 × 5	0.62	*p* < 0.001	0.41 to 0.84	Unblinded

BI: Blinding Index, CI: Confidence Interval. Data are expressed as 95% confidence intervals.

## Data Availability

Tremor data of each participant are available in Supplementary Table S1, including outliers. Moreover, Supplementary Table S2 includes the mean and standard deviations of each variable, including outliers. The datasets generated and analyzed during the current study are available in the Zenodo repository at https://zenodo.org/records/14093286 (accessed on 12 November 2024).

## Supplementary Materials

The following supporting information can be downloaded at https://www.mdpi.com/article/10.3390/jcm13247566/s1; Table S1: Raw data of each participant related to tremor outcomes. Outliers are represented in bold and red numbers; Table S2: Raw data of each secondary outcome (mean and standard deviation), including outliers.
