# Supplementary material for: Transcutaneous Kilohertz High-Frequency Alternating Current at 10 kHz for Upper-Limb Tremor in People with Parkinson’s Disease: A Double-Blind, Randomized, Crossover Study
Source: J Clin Med. 2024 Dec 12;13(24):7566. doi: 10.3390/jcm13247566 (PMC11727874; doi:10.3390/jcm13247566)
Supplement: Supplementary file 1 [file jcm-13-07566-s001.zip › Supplementary Table S1.pdf]

Supplementary Table S1. Raw data of each participant related to tremor outcomes. Outliers are represented in bold and red numbers.

	Rest tremor ($G^2 \times 10^{-3}$)							
	10kHz				Sham			
ID	Pre	During	Post	Post10	Pre	During	Post	Post10
1	81.5	60.3	59.4	82.5	109.0	107.9	95.7	155.8
2	21.7	3.0	6.3	2.5	33.7	17.1	9.1	42.3
3	3.3	2.1	5.3	0.3	1.8	1.7	0.2	0.7
4	0.5	0.5	0.8	1.1	1.3	0.5	0.9	1.4
5	183.4	257.1	60.6	249.1	22.7	89.4	43.0	105.7
6	5.8	2.9	2.2	1.1	9.7	9.8	4.5	0.8
7	0.1	0.1	0.1	0.1	6.1	0.9	1.6	0.5
8	166.3	**537.7**	238.1	113.8	142.8	185.6	99.5	24.6
9	0.1	0.1	0.0	0.0	0.1	0.1	0.1	0.0
10	0.3	0.0	**1019.1**	906.0	95.8	0.2	**831.3**	428.6
11	89.7	68.2	153.2	77.4	65.1	74.5	70.4	119.6
12	100.8	120.0	43.7	51.8	58.2	28.0	8.3	23.2
13	314.4	**543.4**	**1123.6**	1006.1	**1152.9**	**1368.2**	**995.6**	**2246.2**
14	**3034.9**	0.1	0.1	**1573.0**	**1308.9**	208.3	3.7	0.4
15	608.5	214.1	787.5	388.9	48.8	4.3	39.8	530.8
16	1.6	8.1	0.9	1.0	1.2	0.6	1.8	0.5

	Postural tremor ($G^2 \times 10^{-3}$)							
	10kHz				Sham			
ID	Pre	During	Post	Post10	Pre	During	Post	Post10
1	0.3	0.1	0.1	0.1	0.4	0.2	0.2	0.1
2	0.3	0.2	0.2	0.2	0.5	0.2	0.1	0.2
3	0.1	0.3	0.0	0.1	0.1	0.0	0.0	0.1
4	0.0	0.1	0.0	0.1	0.1	0.0	0.1	0.0
5	0.3	0.3	0.1	0.2	0.1	0.1	0.1	0.1
6	2.6	0.3	0.0	0.0	1.1	0.4	0.0	0.0
7	0.0	0.0	0.2	0.0	0.0	0.0	0.0	0.0
8	1.2	1.1	1.6	0.7	0.8	1.9	0.3	0.4
9	**3.8**	0.2	0.2	0.1	**8.2**	1.0	**11.6**	2.0
10	0.3	0.8	0.2	0.1	0.1	0.0	0.1	0.2
11	0.8	0.4	0.5	0.6	0.4	0.4	0.2	0.5
12	3.0	1.1	1.3	1.4	4.8	**4.2**	1.7	1.8
13	0.3	0.6	0.9	0.5	0.6	0.5	0.6	0.2
14	0.6	0.2	0.1	0.3	0.1	0.2	0.1	0.2
15	0.6	0.5	0.4	0.4	0.4	0.5	1.1	0.7
16	3.5	**12.7**	**4.3**	**4.8**	**8.9**	**4.7**	1.2	**2.4**

	Kinetic tremor (G² × 10⁻³)							
	10kHz				Sham			
ID	Pre	During	Post	Post10	Pre	During	Post	Post10
1	2.9	2.0	3.8	2.3	4.4	2.2	3.7	2.5
2	5.0	11.9	3.0	6.3	3.7	3.9	6.1	4.2
3	2.4	4.2	1.8	5.3	1.1	1.0	1.5	3.5
4	1.9	1.1	1.9	1.5	1.8	1.1	2.6	1.2
5	10.5	5.7	13.3	2.1	4.8	5.8	7.2	6.1
6	8.0	2.7	3.5	4.1	6.2	4.1	4.6	**63.6**
7	1.7	2.6	0.4	0.6	0.7	1.7	3.8	9.1
8	6.8	8.0	2.3	3.3	11.6	5.2	4.9	5.4
9	15.0	16.4	3.5	6.8	7.2	12.2	7.8	21.7
10	6.9	8.1	4.0	3.5	3.2	5.3	9.6	3.6
11	5.4	4.0	3.6	2.7	1.6	2.6	1.9	2.2
12	32.3	12.7	6.0	8.5	**64.6**	15.8	10.6	6.5
13	3.0	34.5	1.8	1.4	2.6	2.9	1.3	2.1
14	2.1	5.8	1.7	0.7	0.9	2.8	11.8	3.0
15	6.5	5.5	6.2	6.5	7.5	6.8	2.4	3.5
16	**74.2**	**105.1**	**92.4**	**83.5**	34.1	**56.6**	**60.3**	41.4
