# Supplementary material for: Transcutaneous Kilohertz High-Frequency Alternating Current at 10 kHz for Upper-Limb Tremor in People with Parkinson’s Disease: A Double-Blind, Randomized, Crossover Study
Source: J Clin Med. 2024 Dec 12;13(24):7566. doi: 10.3390/jcm13247566 (PMC11727874; doi:10.3390/jcm13247566)
Supplement: Supplementary file 1 [file jcm-13-07566-s001.zip › Supplementary Table S2.pdf]

Supplementary Table S2. Raw data of each secondary outcome (mean and standard deviation), including outliers.

Outcomes (Units)		10kHz intervention (n=16)	Sham intervention (n=16)
	**Time**	**Mean (SD)**	**Mean (SD)**
**Rest tremor ($G^2 \times 10^{-3}$)**	Pre	288.3 (750.0)	191.1 (409.2)
	During	113.6 (184.9)	131.1 (336.8)
	Post	218.8 (386.8)	137.8 (306.2)
	Post-10	278.4 (469.7)	230.1 (560.8)
**Postural tremor ($G^2 \times 10^{-3}$)**	Pre	1.1 (1.3)	1.7 (2.9)
	During	1.2 (3.1)	0.9 (1.5)
	Post	0.6 (1.1)	1.1 (2.8)
	Post-10	0.6 (1.2)	0.6 (0.8)
**Kinetic tremor ($G^2 \times 10^{-3}$)**	Pre	11.5 (18.3)	9.7 (16.7)
	During	14.4 (25.5)	8.1 (13.5)
	Post	9.3 (22.3)	8.8 (14.1)
	Post-10	8.7 (20.1)	11.2 (17.3)
**Handgrip strength (Kg)**	Pre	23.6 (7.0)	23.1 (7.1)
	During	22.4 (7.3)	23.4 (7.7)
	Post	23.2 (8.0)	23.4 (7.4)
	Post-10	23.8 (7.3)	23.7 (7.3)
**NHPT onset time (s)**	Pre	1.9 (1.1)	1.7 (0.7)
	During	1.9 (0.9)	1.8 (0.9)
	Post	1.6 (1.0)	1.5 (0.6)
	Post-10	1.2 (0.3)	1.4 (0.5)
**NHPT total time (s)**	Pre	37.1 (16.4)	41.4 (30.7)
	During	42.9 (20.1)	43.4 (26.1)
	Post	38.2 (15.9)	38.9 (19.1)
	Post-10	36.4 (17.2)	40.6 (25.7)

NHPT: Nine-Hole Peg Test.
